# Dietary Intake of Soccer Players before, during and after an Official Game: Influence of Competition Level and Playing Position

**DOI:** 10.3390/nu16030337

**Published:** 2024-01-23

**Authors:** Costas Chryssanthopoulos, Athanasios Souglis, Sofia Tsalouhidou, Andrew T. Hulton, Gregory C. Bogdanis, Anatoli Petridou, Anastassios Philippou, Maria Maridaki, Apostolos Theos

**Affiliations:** 1 Department of Physical Education and Sport Science, School of Physical Education and Sport Science, National and Kapodistrian University of Athens, 17237 Athens, Greecegbogdanis@phed.uoa.gr (G.C.B.);; 2 Department of Physiology, Medical School, National and Kapodistrian University of Athens, 11527 Athens, Greece; tfilipou@med.uoa.gr; 3 Laboratory of Evaluation of Human Biological Performance, School of Physical Education and Sport Science at Thessaloniki, Aristotle University of Thessaloniki, 54124 Thessaloniki, Greeceapet@phed.auth.gr (A.P.); 4 Department of Nutrition, Food and Exercise Sciences, Faculty of Health and Medical Sciences, University of Surrey, Guildford GU2 7XH, UK; a.hulton@surrey.ac.uk; 5 Section of Sports Medicine, Department of Community Medicine & Rehabilitation, Umeå University, 901 87 Umeå, Sweden

**Keywords:** dietary survey, football, league level, playing position, official match

## Abstract

Physical demands in soccer differ according to league level and playing position and may influence nutritional requirements. This study examined the effect of competition level and playing position on dietary intake in male soccer players (SP). Diet was weighed and recorded by 123 SP for 3 days; before, on the day, and the day after an official match. SP in the Super League (SL, *n* = 33) division reported higher (*p* < 0.05) average three-day energy (195 ± 36 kJ/kg), carbohydrate (6.0 ± 1.1 g/kg), and protein (2.2 ± 0.5 g/kg) intakes compared to the intakes reported by SP in the 2nd (*n* = 30) (energy: 159 ± 31 kJ/kg; carbohydrate: 4.6 ± 1.2 g/kg; protein: 1.9 ± 0.4 g/kg), 3rd (*n* = 30) (energy: 153 ± 34 kJ/kg; carbohydrate: 4.5 ± 1.2 g/kg; protein: 1.7 ± 0.4 g/kg), and 4th (*n* = 30) (energy: 152 ± 36 kJ/kg; carbohydrate: 4.2 ± 1.2 g/kg; protein: 1.7 ± 0.5 g/kg) national leagues (mean ± SD). Furthermore, when data were analyzed by playing position (pooled data), wide midfielders reported higher (*p* < 0.05) energy (183 ± 33 kJ/kg), carbohydrate (5.4 ± 1.2 g/kg), and fat (1.5 ± 0.4 g/kg) intakes compared to central defenders (energy: 147 ± 37 kJ/kg; carbohydrate: 4.1 ± 1.1 g/kg; fat: 1.2 ± 0.4 g/kg). The dietary intake of SP may differ according to the playing position and competition level, possibly due to different metabolic demands in training and competition.

## 1. Introduction

Soccer is a multifaceted sport that taxes all energy systems due to its high intensity intermitted nature [1]. Soccer could be described as an aerobic sport interspersed with high intensity/velocity actions, due to the main game changing activities contested at a high intensity [2] but embedded onto an aerobic component due to the duration of match play and typical distances covered at a low to moderate intensity [3,4]. However, the overall demands of a soccer match can vary considerably based on a player’s position and tactical role within the team [5]. Conventional distances reported for all outfield players at an elite level are between 11 and 13 km per match, with central midfielders found to cover the greatest distances, and central defenders covering the least [4]. However, the distances covered at a high intensity may be of more importance [2,4]. High intensity distances covered during elite match play have been observed across multiple seasons, with a trend for wide players to complete greater distances at these extreme intensities [6]. In addition to distances covered, Bloomfield and colleagues [7] highlighted that players will also complete more than 1200 unpredictable changes in activity, comprising of approximately 700 turns and 30–40 tackles and jumps, all of which are metabolically and mechanically demanding. 

The level of competition has been identified as a distinguishing characteristic for physical output during match play [8]. Mohr and colleagues [8] observed that players competing at a higher level covered more total distance and significantly more high-speed running (28%) and sprinting (58%) throughout the match. However, as players engage in high-speed events throughout competition, a reduction in these distances completed at higher intensities over time is evident. This highlights the fatiguing nature of soccer throughout competition, in addition to the clear signs of temporal fatigue that are present following intense periods of the game, characterized by periods of reduced power output [8]. Furthermore, significant declines in muscle glycogen have been observed with early pioneering investigations [9] and more recently with robust investigations [10]. Despite the absence of complete depletion of muscle glycogen [10], a significant reduction was observed and accounted for approximately 47% of muscle glycogen stores depleted or almost depleted of glycogen at the end of a non-competitive match with Danish fourth division players. Of further note, following fiber analysis, it was identified that depletion was apparent within type IIa and type IIx fibers, which are responsible for high-intensity actions, and may correlate with the reduction in sprint performance measured. 

The previous literature demonstrates the need to ensure optimal nutritional strategies are a focus to maintain and improve performance during competition. Therefore, approaches to maintain performance and indeed the ability to repeatedly perform high-intensity bouts include the recommendation for a high-CHO diet, due to the association between fatigue and muscle glycogen stores. As training loads and intensity vary across the training week and build up to competition, further CHO strategies have emerged that indicate a periodized approach to CHO feeding is needed [11]. The recommendations for CHO have a broad range from 3 to 8 g/kg depending on the training and fueling requirements, although prior to match day, the recommendations are closely aligned to 6 to 8 g/kg [12]. There is limited data on the nutritional intake of elite soccer players, although findings with a small sample of English Premiership players observed a mean CHO intake during training days of 4.2 ± 1.4 g/kg, increasing to 6.4 ± 2.2 g/kg on match day [13]. These findings correspond with the recommendations set by Collins and colleagues [12] and are higher than recent observations within the Dutch Eredivisie that also saw similar periodized intakes, with increased intake on match day (5.1 ± 1.7 g/kg) compared to rest (3.7 ± 1.7 g/kg) and training (3.9 ± 1.7 g/kg) days [14]. Interestingly, protein intake did vary widely between the two investigations, with the players within the English Premiership averaging a very high intake of 2.4 g/kg [11] compared to a more conservative amount of 1.7 g/kg from the Dutch Eredivisie [14]. These investigations highlight the difference in nutritional intakes between European leagues but have only examined the intake from those players at the highest leagues within their competition and without any specific positional information present. Further analysis from other European leagues with varying levels of competition and positional knowledge may be of worth. 

A further nutritional component for elite soccer players is to ensure adequate recovery following match day, especially during congested fixture periods, which can be common throughout the European leagues. Immediate CHO feeding strategies following competition are recommended to ensure glycogen stores are replenished, with research suggesting that players consume approximately 1–1.2 g/kg in the first few hours following exercise [15,16]. In addition, adequate protein intake is needed to support recovery due to the breakdown of muscle during physical activity. Following whole body exercise, 40 g of protein intake would be more advantageous to simulate muscle protein synthesis and is a critical component of recovery [17]. To the authors’ knowledge, no previous research has investigated the nutritional intake of elite players throughout a competitive match day scenario (examining player preparation from kick off and restoration of glycogen following the final whistle). It has also been noted that nutrition may be a key factor for a lowered immunity in athletes undertaking heavy exercise [18]; therefore, adequate micronutrient intake may warrant attention to ensure players are consuming the recommended intake of key vitamins and minerals to optimize health and performance, alongside more traditional macronutrients.

Therefore, the aim of the current investigation was to quantify the nutritional intake of elite soccer players over a three-day period. Professional and semi-professional soccer players from the Greek Super League and lower divisions were included, with the collection of dietary information commencing the day prior to a competitive match (match day − 1; MD − 1), throughout match day (MD), and culminating the following day (match day + 1; MD + 1). Additional aims of this investigation were to break down the intake throughout match day, to highlight positional differences that may exist, and to explore nutritional intake throughout the lower leagues. 

## 2. Materials and Methods

### 2.1. Experimental Design 

The purpose of the study was to examine the effect of competition level and position of play on the dietary intake of male soccer players (SP) before, during, and after an official match. Male SP were recruited from four different league divisions: Super League (SL), 2nd division, 3rd division, and 4th division. In the first three divisions, the players were professionals, whereas in the 4th division they were semi-professionals. The recruitment was made through coaches after explaining the purpose of the study and the benefit each player would have from his participation. Players were required to weigh and record their diet for 3 days: the day before a formal match, on match day, and the day after a match. A brief description of training was also required, while height and body weight were obtained from recent team records. After excluding under-reporters, dietary record data were analyzed for competition level (SL: *n* = 33; 2nd: *n* = 30; 3rd: *n* = 30; 4th: 30) as well as for 5 playing positions: full backs (FB, *n* = 24), central defenders (CD, *n* = 23), central midfielders (CM, *n* = 24), wide midfielders (WM, *n* = 24), and attackers/strikers (A, *n* = 23). 

### 2.2. Participants

A total of 136 Greek SP were recruited. After exclusion of 13 SP as under-reporters, the diets of 123 SP were considered. When analyzing the data for position of play, the 5 goalkeepers were excluded. All participants were over 18 years of age and were fully informed about the experimental procedures involved before signing a written consent form. The study had the approval of the Ethical Committee of the University, and all procedures were in accordance with the Helsinki Declaration of 1975, as revised in 1996.

### 2.3. Assessment of Under-Reporting

To assess dietary under-reporting, the ratio of energy intake (EI) to basal metabolic rate (BMR) was estimated according to Goldberg et al. [19] as modified by Black [20] for total between-subject variation in physical activity level (PAL), and for within-subject variation in estimated BMR. Energy intake was calculated from dietary records, while BMR was estimated according to Schofield and co-workers [21]. The PAL depends on daily activities and is essential to assign the most appropriate PAL before calculating the cut-offs [20]. Therefore, an individual PAL was assigned to each player calculated as the average PALs for the 3 days of dietary recording. The PALs the day before and after the game day were according to the training soccer players reported and ranged from 1.55 for no training up to 1.94 for training about 45 min at moderate to high (>70% heart rate max) intensity [22]. Since PAL is defined as the ratio of total energy expenditure (TEE) over BMR [18], the PAL for the game day was calculated after estimating TEE, according to Reilly and Thomas [23]. The calculated cut-off value for EI to BMR for the study averaged 1.22 ± 0.004 and the reported average EI to BMR was 1.59 ± 0.04. Thirteen SP were classified as under-reporters and were excluded from further analysis. Their reported EI to BMR averaged 0.91 ± 0.03. 

### 2.4. Procedures

Before participating in the study, the purpose of the study was explained to the players and they were trained on how to properly weigh and record their food intake. Participants weighed (Kenwood chef/major kitchen scale, UK) and recorded their food intake the day before an official game, the match day, as well as the day after competition. Due to practical difficulty in measuring fluids during soccer games, fluids during competition were not recorded. Brand names, food labels, methods of preparation, and recipes for mixed dishes consumed were requested. The time of the meals or snacks before as well as after the game were also recorded. Participants were also required to briefly describe any training sessions the days before and after competition. Food records were inspected within 2 days of completion to ensure proper recordings and to avoid any mistakes in the subsequent analysis. It should be noted that no soccer club had a dietician who monitored the players’ diet. 

### 2.5. Dietary Analysis

Food records were analyzed by a registered dietitian blind to competition level and playing position information. Dietary records were analyzed for macronutrients and micronutrients from food labels and a food database developed in our laboratory based on published data [24,25]. Data were analyzed per day and for an average of the 3-day recording period according to both competition level (*n* = 123) and playing position (*n* = 118, excluding the 5 goalkeepers). Since the ability of an athlete to make the appropriate food selection at the right time is important to improve performance and optimize recovery [26], separate analyses for the pre-game meal consumed about 3–4 h before competition and the food consumed within the first two hours after the game were also made. 

### 2.6. Statistical Analyses

Data were analyzed using SPSS (SPSS, Inc., Chicago, IL, USA v. 22.0). The Shapiro–Wilk test was used to assess normality of data. Data were analyzed by competition/category level and by playing position after excluding the five goalkeepers. Kruskal–Wallis was used for examining basic characteristics, average 3-day macronutrients, and micronutrient intakes at category and playing position level, Friedman one-way analysis of variance (ANOVA) was used for repeated measures for macronutrient intakes per day, irrespective of category/playing position, and two-way ANOVA for independent samples for macronutrient composition of pre-game and recovery (0–2 h post-game) meals. When a significant main effect or interaction was found, simple main effects with Bonferroni adjustment for multiple comparisons were used to locate significant differences between mean values in Kruskal–Wallis and two-way ANOVA, while in the Friedman test, Wilcoxon signed rank test was used as a post hoc method. Also, the effect size (ES) was determined by Hedges’ g (small, <0.3; medium, 0.3–0.8; large, >0.8). Data are reported as mean ± SD, and statistical significance was set at *p* < 0.05.

## 3. Results

### 3.1. Basic Characteristics of Players

Table 1 presents the basic characteristics of players and their soccer experience according to participation level and playing position. A higher (*p* < 0.01) body weight (ES: 0.69–0.95) and BMI (ES: 0.94–1.39) were observed in the SL players compared to the other three leagues, while the players of the 2nd league had more official games played (*p* < 0.05; ES: 0.69–0.94). Also, CD were taller compared to FB, CM, and WM (*p*< 0.05; ES: 0.93–2.39).

### 3.2. Macronutrient Intake by Competition Level

**Figure 1:**
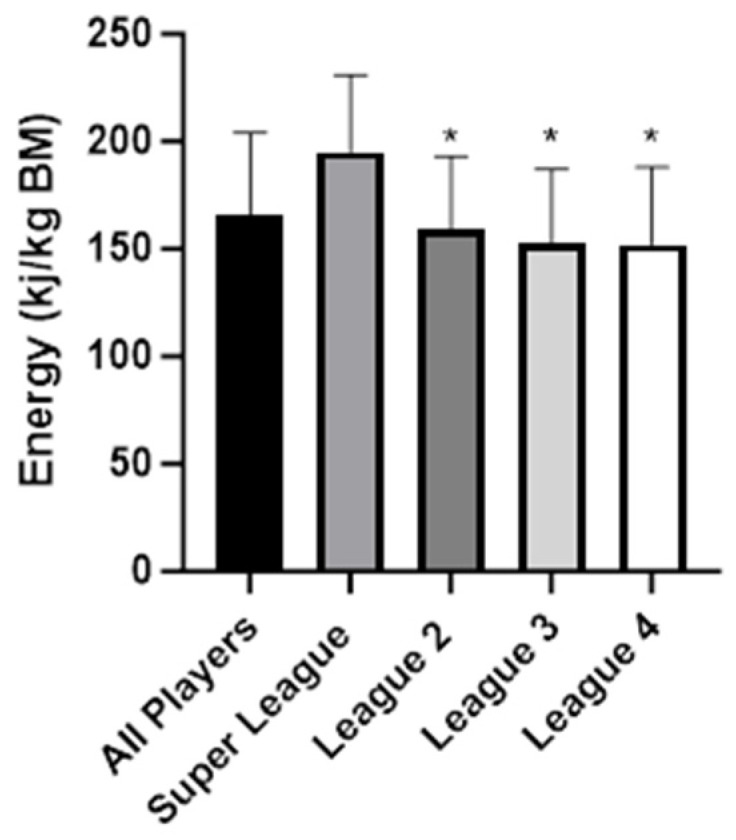
Overall relative energy intake throughout the various league levels. * *p* < 0.05 from Super League.

Table 2 presents the average of the 3 days of macronutrient intake according to competition level. Super league players had higher intakes in energy expressed in MJ and kj/kg (ES: 1.42–1.51) (Figure 1) as well as in CHO (ES: 1.22–1.57) and protein (ES: 0.66–1.10) in g/kg compared to the other three divisions (*p* < 0.05). However, relative contribution of macronutrients to energy intake was not different among different leagues with the exception of a higher fat (ES: 1.04) and lower CHO (ES: 0.94) percentage in the 4th division compared to the SL (*p* < 0.05). 

Table 3 presents the macronutrient intake per day by competition level, while Table S1 in the Supplementary Materials presents the macronutrients per day for all soccer players. Energy in MJ was higher in every day in SL compared to the other three leagues with the exception of day 2 (match day) in the 2nd division (*p* < 0.05; ES: 0.80–1.66). For energy intake in kj/kg and protein in g/kg, SL players had higher intakes on day 3 (the day after the match day), compared to all other divisions (ES for energy: 1.25–1.38; ES for protein: 1.08–1.67), whereas, on day 1 (day before match day), SL players had higher protein intakes (*p* < 0.05; ES: 0.82) compared to the 4th division (Table 3). Also, SL players had higher energy (ES: 0.84–1.14), protein (ES: 0.61–1.09), and fat (ES: 1.18) intakes on day 3 compared to days 1 and 2 (*p* < 0.05). No difference was observed in CHO intake and relative contribution of macronutrients to energy between days and league levels (Table 3). Irrespective of competition level, players had a higher (*p* < 0.05) intake in energy (ES: 0.24–0.32) and fat intake (ES: 0.56) and percentage of fat (ES: 0.37) on day 3 compared to the other two days (Table S1). 

Table 4 presents the data of the pre-game meal composition and the food ingested during the recovery period within the first two hours after the match. No difference was observed in the composition of a pre-game meal between leagues. However, in the 2-h recovery period, energy in MJ (ES: 0.63–1.12) and protein (ES: 0.57–1.15) intake was higher in SL compared to the 3rd and 4th divisions (*p* < 0.05). Energy in kJ/kg (ES: 1.03), protein (ES: 1.15) and fat (ES: 1.00) intake were also higher in SL compared to the 3rd league, while percentage CHO was lower in SL compared to the other divisions (*p* < 0.05; ES: 0.78–1.20) (Table 4). It should be noted that all players in SL ingested some food within two hours after the formal match, whereas four, five, and one player in the 2nd, 3rd, and 4th divisions, respectively, did not eat during that period (Table 4). In terms of alcohol intake, all players avoided alcohol in the pre-game meal, whereas in the recovery period, 14 out of the 33 SP in the SL had alcohol and only 2 in 26, 1 in 25, and 1 in 29 players in the leagues 2, 3, and 4, respectively, consumed alcohol (*p* < 0.05; ES: 0.79–1.00) (Table 4). 

### 3.3. Macronutrient Intake by Playing Position

**Figure 2:**
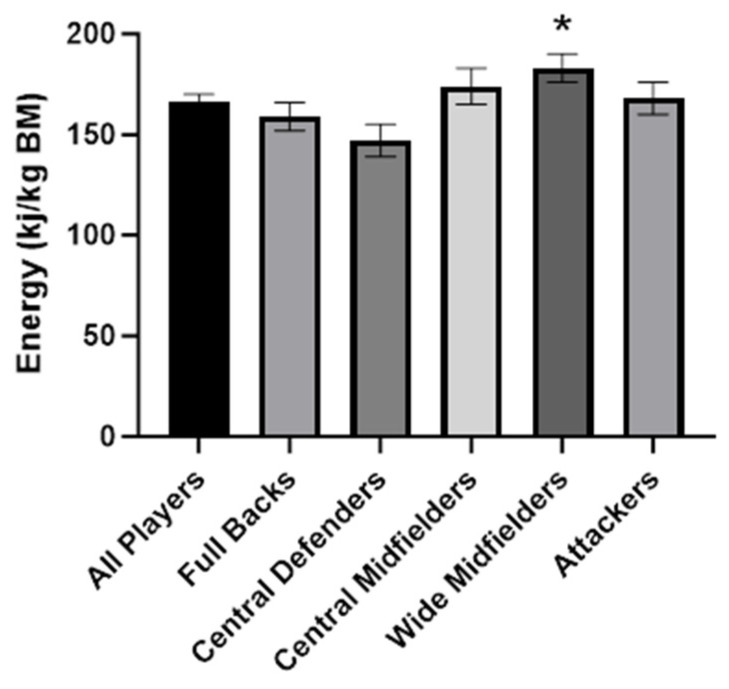
Relative energy intake comparison between playing position. * WM significantly greater than CD (*p* < 0.05).

Table 2 presents the average of the 3 days of macronutrient intake data analyzed according to playing position. Energy (ES: 1.03) (Figure 2), carbohydrate (ES: 1.13), and fat (ES: 0.75) intake per unit of body mass were higher in WM compared to CD, whereas fat intake in WM was also higher (ES: 0.75) compared to FB (*p* < 0.05). 

Table 5 presents the macronutrient intake per day by playing position, while Table S2 presents the macronutrients per day for all these players. No interaction was observed in macronutrient intake between days and position of play (Table 5). Irrespective of playing position, however, energy (ES: 0.33), protein (ES: 0.29), and fat (ES: 0.49) intake relative to body weight was lower in the match day compared to the day after (day 3), while in day 3 the relative contribution of fat to total energy intake (ES: 0.25) was also higher (Table S2; *p* < 0.05). 

Table 6 presents the data of the pre-game meal composition and the food ingested during the recovery period within the first two hours after the game. In terms of the pre-game meal composition, energy intake in kJ relative to body weight was higher in WM compared to CD (ES: 0.92), while carbohydrate intake was also higher in WM (ES: 1.17) and A (ES: 0.98) compared to CD (*p* < 0.05). Energy in kJ/kg (ES: 0.86) and protein intake (ES: 1.00) were also higher in WM and fat intake (ES: 1.00) was higher in CM compared to CD in the recovery period (*p* < 0.05). Regarding alcohol, all players avoided alcohol before the match, whereas in the recovery period, four FB, two CD and A, and five WM and CM consumed alcohol.

### 3.4. Micronutrient Intake

Table S3 in the Supplementary Materials presents the average of the three days of micronutrient intake in the four leagues. With the exception of vitamins A, C, D, and E, cobalamin, biotin, calcium, iodine, and selenium, the intake for all the other micronutrients was higher in SL compared to the other leagues (*p* < 0.05; ES: 0.27–2.03). Only the intake of vitamins D and E were found to be lower than the dietary reference values reported by the European Food Safety Authority [27], whereas for the rest of the micronutrients, the intake was above the EFSA values in all categories (Table S3). 

Table S4 in the Supplementary Materials presents the average of the three days of micronutrient intake according to the playing position. Only vitamin D intake was higher in WM compared to FB (*p* < 0.05; ES: 0.90). No other difference in any micronutrient intake was observed between the different positions. Similarly to the micronutrient intake results by competition level, the micronutrient intake analyzed by playing position showed lower levels in vitamins D and E than the values reported by EFSA, while the intake in all the other micronutrients was above EFSA levels in all playing positions [27] (Table S4).

### 3.5. Dietary Survey Data of Goalkeepers

Table S5 presents the physiological characteristics of the five goalkeepers. Table S6 presents the average of the 3 days of dietary recording of macronutrients for these players and Table S7 presents the micronutrients. 

## 4. Discussion

The main aim of this investigation was to quantify the nutritional intake of elite and sub-elite soccer players over the three-day period around competition, i.e., match day − 1, match day, and match day +1. Players of varying levels of ability were included from the Greek Super League through to Division 4, with the primary findings identifying a significant increased energy intake with SL players averaged over the three days (approximately 800 kcal) compared with their lower division peers, highlighted by greater/significant CHO and protein intake. 

Comparing the three-day nutritional intake, it is clear that SL players gain more energy from all macronutrients, albeit only significant for fat compared to the division 3 players. The difference in energy intake between SL and the lower divisions is characterized by higher amounts of CHO and protein, but with no change to the relative contribution of the macronutrients in total energy intake (i.e., % of CHO, protein, fat). The average CHO intake for the SL players (6.0 ± 1.1 g/kg BM) meets the current recommendations proposed for training and fueling for match day [12], even the guidelines of 5–8 g/kg BM for high training loads (1–3 h/d moderate to high intensity) [28,29]. However, the lower division players fail to meet the CHO targets for match day fueling (Division 2: 4.6 ± 1.2 g/kg BM; Division 3: 4.5 ± 1.2 g/kg BM; Division 4: 4.2 ± 1.2 g/kg BM). This is a finding of interest that may represent the greater training and match load for elite players, requiring greater CHO consumption. Conversely, it could simply show that there is a greater nutritional availability within the club facilities for the SL players, in addition to greater support from medical staff and nutritional awareness, although more research is required to substantiate this. When further scrutinizing the individual days, there is no significant different between the leagues over the three days for CHO, although it is noticeable that the CHO intake for leagues 2, 3, and 4 are very consistent and stay within a narrow range of 4.1–4.6 g/kg BM. When compared to the SL, there is a greater variation of CHO intake with 6.0 ± 1.4 g/kg BM, 5.3 ± 1.2 g/kg BM, and 6.6 ± 1.7 g/kg BM for MD − 1, MD, and MD + 1, respectively. However, again, these higher intakes do not significantly affect the relative amount of each macronutrient, and the higher intake on MD + 1 would support the recovery process, although it must be noted that the fat intake on MD + 1 for SL players is approximately 50 g more than the previous days and would account for an additional 350 kcal, driving the increase in energy intake. Further insights into playing positions identified a positional difference in CHO intake between wide midfielders (5.4 ± 1.2 g/kg BM) and central defenders (4.1 ± 1.1 g/kg BM). This positional difference was the only variation between macronutrient intake but did further result in an increased energy intake of 183 ± 33 kJ/kg BM vs. 147 ± 37 kJ/kg BM for wide midfielders and central defenders, respectively.

The mean protein intake for all players in the current investigation was 1.9 ± 0.5 g/kg BM, which is within the recommended range suggested of 1.6–2.2 g/kg BM needed for training adaptations to occur [28,30], as well as for performing high-volume intense exercise training [29]. The protein intake of the current investigation aligns with that from the Dutch Eredivisie (1.7 g/kg BM) [14]. However, individual league analysis highlights that the average protein intake is significantly higher within the SL players (2.2 ± 0.5 g/kg BM) compared to league 2 (1.9 ± 0.4 g/kg BM), league 3 (1.7 ± 0.4 g/kg BM), and league 4 (1.7 ± 0.5 g/kg BM). Nevertheless, all leagues are within the range previously suggested, and the intake from the SL is more aligned with that from a small sample of players from the English Premiership [13]. It is unclear why there is a difference in protein intake as it is well established that optimal protein intake can be achieved from a mixed diet of whole foods [31]. However, there is evidence suggesting that athletes at a higher level may consume more sports supplements, such as protein, which is widespread throughout football, and its prevalence has been shown to vary according to the sport, training, and performance level [32]. 

Nutritional intake pre-match was also recorded for all players in this investigation. If MD − 1 preparations have been to an optimal standard then muscle glycogen stores should be sufficient and the match day itself may act only as a ‘top up’, and of particular importance to ensure that liver glycogen is fully optimized following reductions due to an overnight fast [33]. Therefore, the CHO recommendations for the pre-match period is 1–3 g/kg BM [12,28]. To the authors’ knowledge, match day intake has not previously been analyzed and broken down to identify pre- and post-match intake within elite soccer players. Findings from players throughout the Greek leagues demonstrates that all players consume an adequate amount of CHO prior to matches with a mean intake of 1.2 ± 0.1 g/kg BM, ranging from 1.0 ± 0.1 to 1.4 ± 0.1 g/kg BM. However, this conflicts with the recommended 2 g/kg BM when examining the periodized CHO approach proposed by Anderson and colleagues [13], highlighting a lack of sufficient CHO for all players. When further examined from a positional sense, clear differences between central defenders to wide midfielders and attackers are observed (Table 6). 

An important aspect of nutrition following competition is the need to refuel, with special interest in CHO and the replenishment of glycogen [34]. Researchers have previously discussed the need to provide CHO immediately, or at least within a two-hour period, following exercise to initiate this replenishment process [15]. Key processes are heightened during the aftermath of exercise to support glycogen resynthesis. Glycogen synthase is activated due to the exercise-induced glycogen use [35], in addition to an increase in insulin sensitivity [36] and enhanced sensitization of the muscle cell membranes to glucose delivery [16]. Players throughout the Greek football leagues all met the requirements purposed for CHO intake to support the replenishment of CHO [11,15,16,28], with a range of CHO intake from 1.1 g/kg BM to 1.4 g/kg BM. The amount of protein consumed in the hours after the match did differ significantly. Players from leagues 3 and 4 consumed significantly less protein compared to the SL players. Again, this finding may represent the availability of nutrition to players post match within the changing rooms, with protein snacks and drinks/shakes the likely source presented to players. Further research into the acute amount of protein provided from the post-match meal/snack may be warranted rather than an overview of the two hours as conducted in the current investigation. The amount consumed by the SL players of 0.8 ± 0.3 g/kg BM during the two hours may be deemed too much if consumed acutely, with reports suggesting that a protein intake of 0.4–0.55 g/kg BM may be the optimal amount to achieve anabolic effects [37]. With regards to the fat intake post match, SL players also consumed significantly more fat compared to league 3 players (0.6 ± 0.3 g/kg BM vs. 0.3 ± 0.3 g/kg BM), and although not to significant levels, the analysis shows that, on average, fat intake was enhanced in the SL player. One finding during the two hours post match that was of interest was that SL players consumed significantly more alcohol than their peers. As mentioned earlier, 14 out of the 33 SL players had alcohol after the game while only one or two consumed alcohol in the other leagues. The SL players consumed 6.0 ± 8.1 g compared to 0.8 ± 2.7, 0.9 ± 3.1, and 0.1 ± 0.6 for players in league 2, 3, and 4, respectively. This may seem quite a lot at first, but in reality, this may suggest that some of the SL players have one alcoholic beverage during this period, whereas it is less likely to occur in the lower leagues. Interestingly, this difference was not observed among the playing positions. Alcohol consumption in soccer is not a novel finding and previous work highlighted that 66% of players in an Italian Serie A declared themselves as regular drinkers of alcoholic beverages [38]. However, a further finding of interest was that alcohol consumption was higher, although not significant, with the wide midfielders compared to other positions and would add to the increased energy intake observed, albeit a redundant energy source. 

Micronutrients are an important nutrient to consider when evaluating the athletic diet. Training and competition add further stress to the metabolic pathways that micronutrients support [39], therefore consideration is required but often overlooked. The importance of such nutrients is due to the essential contribution vitamins and minerals provide with regards to the metabolic processes for energy production, growth, health, and wellbeing. Significant differences are evident between the SL players and the lower division players, although this was expected following the dietary analysis that highlighted the SL players’ superior energy and macronutrient intake. Nevertheless, all players consumed the recommended amounts of B-vitamins, essential for energy metabolism, in addition to other key vitamins such as vitamin C, which agrees with previous research within soccer [40]. The majority of literature advocates meeting the recommended micronutrient intake for general population, without observing any ergogenic effects from higher intakes [28]. However, vitamin E was below the EFSA recommendations [27]. Vitamin E is a fat-soluble antioxidant and a recent review article has suggested that there may be no clear scientific rationale for recommending vitamin E to athletes [41], but we should at least ensure that basic recommendations are met for health purposes. Acute supplementation of vitamin E may play a key role in reducing oxidative stress and supporting competitive performance, but chronic supplementation is best avoided in athletic populations [42]. 

Though data for comparison with soccer players are limited with regard to iron intake [12], iron deficiency has been reported within some athletic populations [43]. However, iron, and other key minerals such as calcium, were also shown to meet the requirements throughout the leagues, suggesting that a well-balanced diet was achieved. Vitamin D is a vitamin of keen interest with muscle function and recovery deemed to decrease with deficiency [44], whilst skeletal health, the prevention of osteoporosis and improved athletic performance are associated with sufficient vitamin D status [45,46]. The recommendations for vitamin D consumption within Europe is 15 µg, or 600 IU [27], although, findings within the current investigation highlight a low vitamin D intake with a mean intake throughout the leagues of 2.5 ± 1.4 µg. These findings are supported within the literature [47,48] and may be of concern, promoting recommendations of vitamin D supplementation amongst players. Vitamin D is unique by the fact that the skin is able to synthesize this nutrient from sunlight, with suggestions that only 20% of our vitamin D production is produced from the diet [49]. However, in spite of the players residing in Greece, the current investigation’s data collection period took place throughout the winter months and enhanced 25-hydroxyvitamin D3 (25[OH]D) production that may have occurred in the summer may not be sufficient to last throughout the winter season. To gain a clear understanding of the vitamin D status, it may be prudent to measure 25[OH]D, which is the primary circulating form of vitamin D, and may give further insight into each player’s needs. 

In practice, these results suggest that greater education regarding the role and importance of CHO is needed in the lower divisions to ensure players are fully aware of the optimal fueling strategies to prepare for competitive soccer matches. This education would need to focus not only on the match’s specific days, but also on the days prior to and following the match for fueling and re-fueling purposes. Further considerations for vitamin D are required, with blood analysis for 25[OH]D recommended to fully understand the players’ vitamin D status correctly. This investigation is notwithstanding its limitations. Recording dietary intake via food records may include an element error, particularly under-reporting [50], although this was felt to be the most appropriate method within this population and timepoint. Whilst the variation of leagues provides a good indication of playing level, there is a lack of data on the actual games themselves, with no match demands provided, both physically and physiologically. This information may support energy intake and certain nutrient intake within the populations and enhance the understanding of the overall intake that may be linked to an increase match load. Further, fluid intake or sports drinks were not recorded during the matches. This is due to the difficulty associated with such measures, as it is difficult to have players measure their fluid intake during competition, although these additional measures would provide a good insight into the hydration practices of these players during competition.

## 5. Conclusions

This investigation is the first to analyze the nutritional intake in elite football players across a range of leagues within the same country around match day. The data highlights an increased nutritional intake by SL players over the three-day period, without a difference in relative contribution of the macronutrients in total energy intake. CHO intake is below the recommendations for competition fueling and recovery for the lower league players and is consumed in a consistent fashion, whereas more variation is observed throughout the three days with SL players. Micronutrient intake met the requirements for all apart from vitamin D, with data suggesting that supplementation of this nutrient is required in the winter months. Finally, the positional analysis demonstrated that wide midfielders have an increased energy and CHO intake compared to the central defenders, likely due to the increase in match load typified by greater overall distance and high-speed distance covered during match play.

## Figures and Tables

**Table 1:** Basic characteristics of players according to category level and playing position (mean ± SD).

Basic Characteristics	Category Level					Playing Position					
	All (*n* = 123)	SL (*n* = 33)	2nd (*n* = 30)	3rd (*n* = 30)	4th (*n* = 30)	All (*n*= 118)	FB (*n* = 24)	CD (*n* = 23)	CM (*n* = 24)	WM (*n* = 24)	A (*n* = 23)
Age (y)	25.7 ± 4.3	25.6 ± 3.8	27.3 ± 4.1	25.4 ± 4.3	24.5 ± 4.5	25.6 ± 4.3	25.3 ± 3.6	26.5 ± 4.8	24.4 ± 4.4	26.2 ± 4.3	25.9 ± 4.4
Weight (kg)	75.7 ± 6.6	79.3 ± 5.7	75.0 ± 6.8 ^1^	73.7 ± 6.1 ^1^	74.5 ± 6.0 ^1^	75.3 ± 6.3	71.8 ± 4.0 ^3^	80.1 ± 6.4	75.3 ± 7.5	73.7 ± 5.4 ^3^	76.0 ± 5.2 ^4^
Height (cm)	180.2 ± 5.5	181.1 ± 5.5	181.1 ± 6.2	179.4 ± 4.3	179.1 ± 5.2	180.0 ± 5.3	176.2 ± 2.9 ^3^	185.2 ± 4.5	179.0 ± 6.2 ^3^	178.0 ± 3.7 ^3^	181.6 ± 3.9 ^4^
BMI (kg/m^2^)	23.3 ± 1.2	24.2 ± 0.8	22.8 ± 1.2 ^1^	22.9 ± 1.1 ^1^	23.2 ± 1.3 ^1^	23.2 ± 1.2	23.1 ± 1.1	23.3 ± 1.0	23.5 ± 1.1	23.3 ± 1.5	23.0 ± 1.2
Years of Playing Soccer (y)	9.5 ± 3.8	9.7 ± 3.2	10.4 ± 2.9	9.0 ± 4.0	8.9 ± 4.6	9.5 ± 3.8	8.7 ± 3.3	10.4 ± 3.4	9.0 ± 4.4	9.8 ± 3.7	9.7 ± 4.2
Official Games Played	190 ± 122	158 ± 100 ^2^	256 ± 108	175 ± 126 ^2^	173 ± 127 ^2^	193 ± 123	189 ± 98	224 ± 145	170 ± 133	192 ± 116	189 ± 123

A = attackers/strikers; BMI = body mass index; CD = central defenders; CM = central midfielders; FB = full backs; SL = Super League; WM = wide midfielders. ^1^ Different from SL (*p* < 0.01); ^2^ Different from 2nd category (*p* < 0.05); ^3^ Different from CD (*p* < 0.05); ^4^ Different from FB (*p* < 0.05).

**Table 2:** Average of 3 days of macronutrient intake according to category level and playing position (mean ± SD).

	Category Level					Playing Position					
	All (*n* = 123)	SL (*n* = 33)	2nd (*n* = 30)	3rd (*n* = 30)	4th (*n* = 30)	All (*n*= 118)	FB (*n* = 24)	CD (*n* = 23)	CM (*n* = 24)	WM (*n* = 24)	A (*n* = 23)
Energy (MJ)	12.5 ± 3.2	15.3 ± 2.6	11.8 ± 2.3 ^1^	11.3 ± 2.9 ^1^	11.3 ± 2.7 ^1^	12.6 ± 3.2	11.5 ± 2.6	11.7 ± 2.9	13.1 ± 3.8	13.6 ± 2.9	12.8 ± 3.2
Energy (kcal)	2988 ± 765	3657 ± 621	2820 ± 550 ^1^	2701 ± 693 ^1^	2702 ± 645 ^1^	3011 ± 765	2749 ± 621	2796 ± 693	3131 ± 908	3250 ± 693	3059 ± 765
CHO (g/kg BM)	4.8 ± 1.4	6.0 ± 1.1	4.6 ± 1.2 ^1^	4.5 ± 1.2 ^1^	4.2 ± 1.2 ^1^	4.9 ± 1.4	4.9 ± 1.0	4.1 ± 1.1	5.1 ± 1.8	5.4 ± 1.2 ^2^	4.9 ± 1.4
Protein (g/kg BM)	1.9 ± 0.5	2.2 ± 0.5	1.9 ± 0.4 ^1^	1.7 ± 0.4 ^1^	1.7 ± 0.5 ^1^	1.9 ± 0.5	1.8 ± 0.5	1.8 ± 0.5	1.9 ± 0.5	2.0 ± 0.4	2.0 ± 0.5
Fat (g/kg BM)	1.4 ± 0.4	1.5 ± 0.3	1.3 ± 0.3	1.3 ± 0.4 ^1^	1.4 ± 0.5	1.4 ± 0.4	1.2 ± 0.4 ^3^	1.2 ± 0.4	1.5 ± 0.3	1.5 ± 0.4 ^2^	1.4 ± 0.4
% CHO	49 ± 6	51 ± 3	48 ± 6	49 ± 6	46 ± 7 ^1^	49 ± 6	51 ± 5	47 ± 5	49 ± 7	49 ± 7	48 ± 6
% Protein	19 ± 4	19 ± 2	20 ± 3	20 ± 4	19 ± 5	19 ± 3	19 ± 4	21 ± 3	19 ± 3	18 ± 3	20 ± 3
% Fat	31 ± 5	29 ± 3	32 ± 5	31 ± 6	34 ± 6 ^1^	31 ± 5	29 ± 5	31 ± 5	32 ± 6	32 ± 6	32 ± 5
% Alcohol	0.7 ± 1.1	0.7 ± 0.8	0.6 ± 1.1	0.4 ± 0.8	0.9 ± 1.5	0.7 ± 1.1	0.5 ± 1.0	0.6 ± 1.1	0.5 ± 0.6	1.2 ± 1.6	0.5 ± 1.0
Fiber (g/1000 kcal)	6.5 ± 1.8	7.1 ± 1.2	6.5 ± 2.1	6.5 ± 1.8	5.6 ± 1.6 ^1^	6.5 ± 1.8	6.7 ± 2.0	6.6 ± 2.0	6.1 ± 1.7	6.6 ± 1.6	6.6 ± 1.8
% Saturated	12 ± 3	11 ± 2	12 ± 3 ^1^	12 ± 3	13 ± 3 ^1^	12 ± 3	12 ± 3	12 ± 3	13 ± 3	12 ± 3	12 ± 3
% Monoun-saturated	11 ± 3	10 ± 2	11 ± 2	11 ± 2	12 ± 3 ^1^	11 ± 2	10 ± 2	11 ± 2	11 ± 2	11 ± 3	11 ± 2
% Polyun-saturated	4 ± 1	3 ± 0.7	4 ± 1.1 ^1^	4 ± 1.3	5 ± 1.8 ^1^	4 ± 1	4 ± 1	4 ± 1	4 ± 1	4 ± 2	4 ± 1

A = attackers/strikers; BM = body mass; CD = central defenders; CHO = carbohydrate; CM = central midfielders; FB = full backs; SL = Super League; WM = wide midfielders. ^1^ Different from SL (*p* < 0.05); ^2^ Different from CD (*p* < 0.05); ^3^ Different from WM (*p* < 0.05).

**Table 3:** Macronutrient intake during the 3-day recording period for the four categories (mean ± SD).

	SL (*n* = 33)			2nd Category (*n* = 30)			3rd Category (*n* = 30)			4th Category (*n* = 30)		
	Day 1	Day 2 (Match Day)	Day 3	Day 1	Day 2 (Match Day)	Day 3	Day 1	Day 2 (Match Day)	Day 3	Day 1	Day 2 (Match Day)	Day 3
Energy (MJ)	14.6 ± 3.2 ^2^	13.6 ± 2.6 ^2^	17.8 ± 3.9	11.9 ± 3.2 ^1^	11.8 ± 2.9	11.8 ± 3.7 ^1^	11.4 ± 3.6 ^1^	11.0 ± 3.6 ^1^	11.5 ± 4.0 ^1^	11.2 ± 3.4 ^1^	11.2 ± 3.4 ^1^	11.5 ± 3.7 ^1^
Energy (kcal)	3489 ± 765 ^2^	3250 ± 621 ^2^	4254 ± 932	2844 ± 765 ^1^	2820 ± 693	2820 ± 884 ^1^	2725 ± 860 ^1^	2629 ± 860 ^1^	2749 ± 956 ^1^	2677 ± 813 ^1^	2677 ± 813 ^1^	2749 ± 884 ^1^
Energy (kj/Kg BM)	185 ± 42 ^2^	173 ± 36 ^2^	226 ± 55	158 ± 37	158 ± 40	159 ± 52 ^1^	154 ± 45 ^1^	150 ± 38	155 ± 49 ^1^	151 ± 47 ^1^	151 ± 45	155 ± 47 ^1^
CHO (g/Kg BM)	6.0 ± 1.4	5.3 ± 1.2	6.6 ± 1.7	4.5 ± 1.3	4.6 ± 1.5	4.5 ± 1.8	4.4 ± 1.7	4.5 ± 1.4	4.5 ± 1.7	4.4 ± 1.4	4.2 ± 1.4	4.1 ± 1.5
Protein (g/Kg BM)	2.2 ± 0.7 ^2^	2.0 ± 0.5 ^2^	2.6 ± 0.6	1.8 ± 0.5	1.9 ± 0.6	1.9 ± 0.7 ^1^	1.9 ± 0.6	1.7 ± 0.5	1.6 ± 0.6 ^1^	1.7 ± 0.5 ^1^	1.7 ± 0.6	1.7 ± 0.5 ^1^
Fat (g/Kg BM)	1.3 ± 0.4 ^2^	1.3 ± 0.4 ^2^	1.9 ± 0.6	1.4 ± 0.5	1.3 ± 0.4	1.3 ± 0.6 ^1^	1.3 ± 0.4	1.2 ± 0.5	1.4 ± 0.6 ^1^	1.3 ± 0.6	1.3 ± 0.6	1.5 ± 0.8
% CHO	54 ± 6	51 ± 7	50 ± 6	48 ± 6	48 ± 9	47 ± 9	47 ± 9	51 ± 9	49 ± 8	49 ± 9	47 ± 11	45 ± 11
% Protein	20 ± 4	19 ± 3	19 ± 3	20 ± 5	20 ± 5	21 ± 7	21 ± 6	20 ± 5	18 ± 7	19 ± 4	19 ± 8	19 ± 6
% Fat	26 ± 4	29 ± 6	31 ± 6	32 ± 7	31 ± 7	32 ± 9	32 ± 7	29 ± 7	33 ± 10	31 ± 8	33 ± 8	35 ± 10

BM = body mass; SL = Super League; ^1^ Different from Super League (*p* < 0.05); ^2^ Different from day 3 (*p* < 0.05).

**Table 4:** Macronutrient intake of pre-game meal and post-game (0–2 h) recovery period after the formal match for the four categories (mean ± SD).

	Pre-Game Meal (*n* = 123)					Recovery Period 0–2 h (*n* = 113)				
	All Subjects	SL (*n* = 33)	2nd Category (*n* = 30)	3rd Category (*n* = 30)	4th Category (*n* = 30)	All Subjects	SL (*n* = 33)	2nd Category (*n* = 26)	3rd Category (*n* = 25)	4th Category (*n* = 29)
Energy (MJ)	3.1 ± 1.3	3.6 ± 1.4	3.1 ± 1.1	3.0 ± 1.2	2.8 ± 1.5	3.8 ± 2.3	4.8 ± 2.0	3.9 ± 2.9	2.7 ± 1.7 ^1^	3.5 ± 2.1 ^1^
Energy (kcal)	741 ± 311	860 ± 335	741 ± 263	717 ± 287	669 ± 359	908 ± 550	1147 ± 478	932 ± 693	645 ± 406 ^1^	837 ± 502 ^1^
Energy (Kj/Kg BM)	41 ± 18	45 ± 18	41 ± 14	40 ± 17	38 ± 20	50 ± 30	61 ± 25	51 ± 38	36 ± 23 ^1^	47 ± 29
CHO (g/Kg BM)	1.2 ± 0.6	1.4 ± 0.7	1.1 ± 0.5	1.3 ± 0.7	1.0 ± 0.5	1.3 ± 0.7	1.3 ± 0.6	1.4 ± 1.0	1.1 ± 0.6	1.2 ± 0.7
Protein (g/Kg BM)	0.6 ± 0.5	0.6 ± 0.4	0.6 ± 0.3	0.5 ± 0.3	0.5 ± 0.8	0.6 ± 0.4	0.8 ± 0.3	0.6 ± 0.6	0.4 ± 0.4 ^1^	0.6 ± 0.4 ^1^
Fat (g/Kg BM)	0.3 ± 0.2	0.3 ± 0.2	0.4 ± 0.2	0.3 ± 0.2	0.3 ± 0.2	0.5 ± 0.4	0.6 ± 0.3	0.5 ± 0.4	0.3 ± 0.3 ^1^	0.5 ± 0.5
Alcohol (g)	0	0	0	0	0	2.2 ± 5.4	6.0 ± 8.1	0.8 ± 2.7 ^1^	0.9 ± 3.1 ^1^	0.1 ± 0.6 ^1^
% CHO	50 ± 17	53 ± 13	45 ± 15	52 ± 19	50 ± 19	50 ± 22	37 ± 10	57 ± 23 ^1^	58 ± 24 ^1^	51 ± 24 ^1^
% Protein	22 ± 12	24 ± 11	24 ± 11	20 ± 9	21 ± 16	19 ± 11	23 ± 7	16 ± 10 ^1^	17 ± 13 ^1^	19 ± 13
% Fat	28 ± 13	23 ± 11	31 ± 14	28 ± 14	29 ± 11	30 ± 16	37 ± 9	27 ± 17	24 ± 15 ^1^	30 ± 19
% Alcohol	0	0	0	0	0	1 ± 3	3 ± 4	0.4 ± 1.4 ^1^	0.7 ± 2.3 ^1^	0.1 ± 0.5 ^1^

BM = body mass; CHO = carbohydrate; SL = Super League; ^1^ Different from Super League (*p* < 0.05).

**Table 5:** Macronutrient intake during the 3-day recording period for the five playing positions (mean ± SD).

	FB (*n* = 24)			CD (*n* = 23)			CM (*n* = 24)			WM (*n* = 24)			A (*n* = 23)		
	Day 1	Day 2 (Match Day)	Day 3	Day 1	Day 2 (Match Day)	Day 3	Day 1	Day 2 (Match Day)	Day 3	Day 1	Day 2 (Match Day)	Day 3	Day 1	Day 2 (Match Day)	Day 3
Energy (MJ)	10.3 ± 2.9	11.7 ± 2.7	12.5 ± 4.4	11.5 ± 3.4	11.8 ± 3.5	11.8 ± 3.9	13.2 ± 4.0	12.1 ± 3.2	14.0 ± 6.2	13.5 ± 3.0	12.5 ± 3.6	14.8 ± 3.7	12.6 ± 3.2	12.1 ± 3.3	13.8 ± 5.1
Energy (kcal)	2462 ± 693	2796 ± 645	2988 ± 1052	2749 ± 813	2820 ± 837	2820 ± 932	3155 ± 956	2892 ± 765	3346 ± 1482	3227 ± 717	2988 ± 860	3537 ± 884	3011 ± 765	2892 ± 789	3298 ± 1219
Energy (KJ/Kg BM)	143 ± 37	163 ± 35	172 ± 56	144 ± 42	148 ± 47	147 ± 46	176 ± 51	160 ± 40	185 ± 78	183 ± 39	168 ± 45	199 ± 43	165 ± 40	159 ± 39	181 ± 62
CHO (g/Kg BM)	4.3 ± 1.5	4.8 ± 1.1	5.4 ± 1.6	4.1 ± 1.3	4.3 ± 1.5	4.0 ± 1.3	5.3 ± 1.9	4.8 ± 1.6	5.4 ± 2.6	5.5 ± 1.4	5.1 ± 1.5	5.5 ± 1.7	5.0 ± 1.5	4.6 ± 1.3	5.0 ± 2.1
Protein (g/Kg BM)	1.7 ± 0.5	2.0 ± 1.1	1.8 ± 0.7	1.9 ± 0.7	1.7 ± 0.6	1.8 ± 0.5	2.0 ± 0.7	1.8 ± 0.5	2.0 ± 0.8	2.0 ± 0.6	1.7 ± 0.5	2.3 ± 0.6	1.9 ± 0.5	1.9 ± 0.7	2.1 ± 0.7
Fat (g/Kg BM)	1.1 ± 0.3	1.3 ± 0.5	1.4 ± 0.7	1.2 ± 0.5	1.2 ± 0.5	1.3 ± 0.7	1.4 ± 0.5	1.3 ± 0.4	1.6 ± 0.8	1.5 ± 0.6	1.4 ± 0.6	1.8 ± 0.6	1.3 ± 0.4	1.3 ± 0.5	1.6 ± 0.7
% CHO	50 ± 9	50 ± 10	54 ± 8	48 ± 9	49 ± 9	45 ± 9	50 ± 8	49 ± 9	48 ± 9	51 ± 8	51 ± 8	46 ± 9	50 ± 7	49 ± 9	46 ± 7
% Protein	20 ± 5	20 ± 7	17 ± 4	22 ± 5	20 ± 4	21 ± 6	19 ± 5	20 ± 5	19 ± 5	19 ± 4	18 ± 4	19 ± 5	20 ± 4	20 ± 5	20 ± 7
% Fat	29 ± 7	29 ± 7	29 ± 8	30 ± 7	30 ± 7	33 ± 10	31 ± 8	30 ± 7	33 ± 8	30 ± 8	30 ± 6	34 ± 9	30 ± 6	30 ± 7	33 ± 7

A = attackers/strikers; BM = body mass; CD = central defenders; CHO = carbohydrate; CM = central midfielders; FB = full backs; WM = wide midfielders.

**Table 6:** Macronutrient intake of pre-game meal and post-game (0–2 h) recovery period after the formal match for the five playing positions (mean ± SD).

	Pre-Game Meal (*n* = 118)						Recovery Period 0–2 h (*n* = 108)					
	All Subjects	FB (*n* = 24)	CD (*n* = 23)	CM (*n* = 24)	WM (*n* = 24)	A (*n* = 23)	All Subjects	FB (*n* = 23)	CD (*n* = 22)	CM (*n* = 19)	WM (*n* = 23)	A (*n* = 21)
Energy (MJ)	3.1 ± 1.3	2.9 ± 1.4	2.6 ± 1.0	3.3 ± 1.5	3.5 ± 1.2	3.3 ± 1.3	3.8 ± 2.3	3.1 ± 2.0	2.9 ± 2.3	4.3 ± 1.9 ^1^	4.9 ± 2.7	3.8 ± 2.4
Energy (kcal)	741 ± 311	693 ± 335	621 ± 239	789 ± 359	837 ± 287	789 ± 311	908 ± 550	741 ± 478	693 ± 550	1028 ± 454 ^1^	1171 ± 645	908 ± 574
Energy (KJ/Kg BM)	42 ± 18	40 ± 20	33 ± 13	44 ± 18	47 ± 17 ^1^	44 ± 18	50 ± 31	43 ± 27	37 ± 30	57 ± 25 ^1^	65 ± 35 ^1^	48 ± 29
CHO (g/Kg BM)	1.2 ± 0.6	1.1 ± 0.7	0.9 ± 0.4	1.3 ± 0.6	1.5 ± 0.6 ^1^	1.4 ± 0.6 ^1^	1.3 ± 0.7	1.1 ± 0.7	1.1 ± 0.9	1.4 ± 0.6	1.6 ± 0.7	1.2 ± 0.6
Protein (g/Kg BM)	0.6 ± 0.5	0.6 ± 0.8	0.4 ± 0.2	0.5 ± 0.3	0.6 ± 0.4	0.6 ± 0.3	0.6 ± 0.4	0.6 ± 0.5	0.4 ± 0.4	0.6 ± 0.3	0.8 ± 0.4	0.6 ± 0.5
Fat (g/Kg BM)	0.3 ± 0.2	0.3 ± 0.2	0.3 ± 0.2	0.4 ± 0.2	0.3 ± 0.2	0.3 ± 0.2	0.5 ± 0.4	0.4 ± 0.3	0.3 ± 0.3	0.6 ± 0.3 ^1^	0.6 ± 0.5	0.5 ± 0.4
Alcohol (g)	0	0	0	0	0	0	2.3 ± 5.5	3.2 ± 6.8	0.6 ± 2.0	2.7 ± 4.7	3.4 ± 7.3	1.4 ± 4.6
% CHO	51 ± 16	49 ± 19	44 ± 15	50 ± 15	55 ± 16	56 ± 14	51 ± 22	52 ± 26	58 ± 26	46 ± 16	46 ± 13	53 ± 23
% Protein	22 ± 12	23 ± 14	24 ± 14	21 ± 9	20 ± 10	21 ± 11	19 ± 11	19 ± 11	16 ± 12	20 ± 12	20 ± 8	17 ± 11
% Fat	28 ± 13	28 ± 9	32 ± 16	30 ± 16	25 ± 11	23 ± 10	30 ± 16	27 ± 19	25 ± 18	32 ± 14	33 ± 15	30 ± 16
% Alcohol	0	0	0	0	0	0	1 ± 3	2 ± 4	1 ± 2	2 ± 3	1 ± 3	1 ± 3

A = attackers/strikers; BM = body mass; CD = central defenders; CHO = carbohydrate; CM = central midfielders; FB = full backs; WM = wide midfielders. ^1^ Different from CD (*p* < 0.05).

## Data Availability

Data are contained within the article and Supplementary Materials.

## Supplementary Materials

The following supporting information can be downloaded at: https://www.mdpi.com/article/10.3390/nu16030337/s1, Table S1: Macronutrient intake during the 3-day recording period for all (n = 123) subjects (mean ± SD); Table S2: Macronutrient intake during the 3-day recording period for all (n = 118) subjects excluding the goalkeepers (mean ± SD); Table S3: Average of the 3-day micronutrient intake in the four categories. The numbers in parentheses indicate the percentage of the Dietary Reference Values by the European Food Safety Authority (mean ± SD); Table S4: Average of the 3-day micronutrient intake in the five playing positions excluding the goalkeepers. The numbers in parentheses indicate the percentage of the Dietary Reference Values by the European Food Safety Authority (mean ± SD); Table S5: Basic characteristics of the five goalkeepers (mean ± SD); Table S6: Macronutrient intake during the 3-day recording period for the five goalkeepers (mean ± SD); Table S7: Average of the 3-day micronutrient intake in the five goalkeepers. The numbers in parentheses indicate the percentage of the Dietary Reference Values by the European Food Safety Authority (mean ± SD).
