# Supplementary material for: Dietary Intake of Soccer Players before, during and after an Official Game: Influence of Competition Level and Playing Position
Source: Nutrients. 2024 Jan 23;16(3):337. doi: 10.3390/nu16030337 (PMC10856869; doi:10.3390/nu16030337)
Supplement: Supplementary file 1 [file nutrients-16-00337-s001.zip › nutrients-2797350-supplementary.pdf]

Dietary intake of soccer players before, during and after an official game: Influence of competition level and playing position

Costas Chryssanthopoulos [1, 2, *], Athanasios Souglis [1], Sofia Tsalouhidou [3], Andrew T. Hulton [4], Gregory C. Bogdanis[1], Anatoli Petridou [3], Anastassios Philippou [2], Maria Maridaki [1], and Apostolos Theos [5]

[1] Department of Physical Education and Sport Science, School of Physical Education and Sport Science, National and Kapodistrian University of Athens, 17237 Athens, Greece

[2] Department of Physiology, Medical School, National and Kapodistrian University of Athens, 11527 Athens, Greece

[3] Laboratory of Evaluation of Human Biological Performance, School of Physical Education and Sports Science at Thessaloniki, Aristotle University of Thessaloniki, 54124 Thessaloniki, Greece

[4] Department of Nutrition, Food and Exercise Sciences, Faculty of Health and Medical Sciences, University of Surrey, Guildford GU2 7XH, UK

[5] Section of Sports Medicine, Department of Community Medicine & Rehabilitation, Umeå University, 901 87 Umeå, Sweden

*Correspondence: chryssan@phed.uoa.gr; Tel.: +30-210-7462612

Table S1: Macronutrient intake during the 3-day recording period for all (n=123) subjects (mean ± SD) .

Macronutrient	Day 1	Day 2 (Match Day)	Day 3
Energy (MJ)	12.3 ± 3.6[1]	12.0 ± 3.2[1]	13.3 ± 4.8
Energy (kcal)	2940 ± 860[1]	2868 ± 765[1]	3179 ± 1147
Energy (Kj/Kg BM)	163 ± 45[1]	158 ± 41[1]	175 ± 60
CHO (g/Kg BM)	4.9 ± 1.6	4.7 ± 1.4	5.0 ± 2.0
Protein (g/Kg BM)	1.9 ± 0.6[2]	1.8 ± 0.7[1]	2.0 ± 0.7
Fat (g/Kg BM)	1.3 ± 0.5[1]	1.3 ± 0.5[1]	1.5 ± 0.06
% CHO	50 ± 8	50 ± 9	48 ± 9
% Protein	20 ± 5	20 ± 5	19 ± 6
% Fat	30 ± 7[1]	30 ± 7[1]	33 ± 9

[1]Different from Day 3 ($p < 0.05$); [2]Different from Day 2 ($p < 0.05$).

Table S2: Macronutrient intake during the 3-day recording period for all (n= 118) subjects excluding the goalkeepers (mean ± SD).

Macronutrient	Day 1	Day 2 (Match Day)	Day 3
Energy (MJ)	12.2 ± 3.5[1]	12.0 ± 3.2[1]	13.4 ± 4.8
Energy (kcal)	2916 ± 837[1]	2868 ± 765[1]	3203 ± 1147
Energy (Kj/Kg BM)	162 ± 45[1]	160 ± 41[1]	177 ± 60
CHO (g/Kg BM)	4.9 ± 1.6	4.7 ± 1.4	5.1 ± 2.0
Protein (g/Kg BM)	1.9 ± 0.6	1.8 ± 0.7[1]	2.0 ± 0.7
Fat (g/Kg BM)	1.3 ± 0.5[1]	1.3 ± 0.5[1]	1.6 ± 0.7
% CHO	50 ± 8	50 ± 9[1]	48 ± 9
% Protein	20 ± 5	19 ± 5	19 ± 6
% Fat	30 ± 7[1]	30 ± 7[1]	32 ± 9

[1]Different from Day 3 ($p < 0.05$)

Table S3: Average of the 3-day micronutrient intake in the four categories.
The numbers in parentheses indicate the percentage of the Dietary Reference Values by the European Food Safety Authority (mean ± SD).

Micronutrient	All Subjects (n=123)	Super League (n=33)	2nd Category (n=30)	3rd Category (n=30)	4th Category (n=30)
Vitamin A (μg)	1006 ± 1009 (134%)	860 ± 419 (115%)	920 ± 508 (123%)	963 ± 686 (128%)	1306 ± 1811 (174%)
Thiamin (mg/MJ)	0.24 ± 0.08 (240%)	0.25 ± 0.07 (250%)	0.23 ± 0.08[1] (230%)	0.23 ± 0.08[1] (230%)	0.23 ± 0.08[1] (230%)
Riboflavin (mg)	2.5 ± 0.8 (156%)	3.0 ± 0.6 (188%)	2.5 ± 0.8[1] (156%)	2.1 ± 0.7[1] (131%)	2.2 ± 0.7[1] (138%)
Niacin (mg/MJ)	3.4 ± 1.0 (213%)	3.7 ± 0.6 (231%)	3.4 ± 0.9[1] (213)	3.4 ± 1.3[1] (213%)	3.1 ± 1.0[1] (194%)
Pantothenic Acid (mg)	8.1 ± 2.6 (162%)	10.1 ± 2.2 (202%)	7.8 ± 1.9[1] (156%)	7.5 ± 2.8[1] (150%)	6.8 ± 1.8[1] (136%)
Vitamin B6 (mg)	3.6 ± 1.2 (212%)	4.6 ± 1.1 (271%)	3.4 ± 1.1[1] (200%)	3.2 ± 1.1[1] (188%)	3.1 ± 0.9[1] (182%)
Biotin (μg)	43 ± 17 (108%)	55 ± 13 (138%)	45 ± 14 (113%)	37 ± 20[1, 2] (93%)	35 ± 13[1, 2] (88%)
Folate (μg)	461 ± 177 (140%)	608 ± 112 (184%)	450 ± 176[1] (136%)	381 ± 158[1] (115%)	388 ± 151[1] (118%)
Cobalamin (μg)	7.8 ± 3.1 (195%)	8.3 ± 1.8 (208%)	7.7 ± 3.0 (193%)	6.8 ± 2.6[1] (170%)	7.8 ± 4.4 (195%)
Vitamin C (mg)	159 ± 112 (145%)	206 ± 117 (187%)	147 ± 138[1] (134%)	147 ± 101 (134%)	132 ± 61 (120%)
Vitamin D (μg)	2.5 ± 1.4 (17%)	2.9 ± 1.2 (19%)	2.9 ± 1.5 (19%)	2.0 ± 0.9[1] (13%)	2.4 ± 1.6 (16%)
Vitamin E (mg)	10.4 ± 4.5 (80%)	12.0 ± 3.7 (92%)	10.6 ± 4.2 (82%)	9.6 ± 4.4 (74%)	9.4 ± 5.0[1] (72%)
Calcium (mg)	1293± 352 (136%)	1489± 294 (157%)	1291± 358 (136%)	1242± 327[1] (131%)	1131± 320[1] (119%)
Iron (mg)	17 ± 5 (155%)	22 ± 4 (200%)	16 ± 5[1] (145%)	15 ± 5[1] (136%)	15 ± 5[1] (136%)
Sodium (mg)	4142 ± 1597 (216%)	5565 ± 1574 (278%)	3529 ± 1081[1] (177%)	3684 ± 1535[1] (184%)	3648 ± 1280[1] (182%)
Potassium (mg)	4322 ± 1272 (124%)	5562 ± 1006 (159%)	4129 ± 1041[1] (118%)	3823 ± 1039[1] (105%)	3649 ± 934[1] (104%)
Magnesium (mg)	406 ± 126 (116%)	517 ± 103 (148%)	395 ± 107[1] (113%)	369 ± 118[1] (105%)	332 ± 85[1] (95%)
Phosphorus (mg)	2091 ± 573 (380%)	2601 ± 446 (473%)	2055 ± 512[1] (374%)	1915 ± 512[1] (348%)	1745 ± 391[1] (317%)
Copper (mg)	1.8 ± 1.0 (113%)	2.3 ± 0.8 (144%)	1.5 ± 0.4[1] (94%)	1.7 ± 0.7[1] (107%)	1.9 ± 1.7[1] (119%)
Zinc (mg)	16 ± 5 (114%)	20 ± 4 (143%)	16 ± 4[1] (114%)	15 ± 5[1] (107%)	15 ± 5[1] (107%)
Chloride (mg)	6805 ± 2619 (220%)	9191 ± 2502 (296%)	5866 ± 1785[1] (189%)	5966 ± 2204[1] (193%)	5956 ± 2094[1] (192%)
Manganese (mg)	4.5 ± 2.1 (150%)	6.2 ± 2.0 (207%)	4.3 ± 2.0[1] (143%)	4.1 ± 1.7[1] (137%)	3.4 ± 1.3[1] (113%)
Iodine (μg)	211 ± 95 (141%)	240 ± 92 (160%)	203 ± 89 (135%)	208 ± 102 (139%)	189 ± 85 (126%)
Selenium (μg)	79 ± 25 (113%)	88 ± 21 (126%)	77 ± 17 (110%)	75 ± 24 (107%)	76 ± 33[1] (109%)

[1]Different from Super League ($p < 0.05$); [2]Different from 2nd Category ($p < 0.05$)

Table S4: Average of the 3-day micronutrient intake in the five playing positions excluding the goalkeepers. The numbers in parentheses indicate the percentage of the Dietary Reference Values by the European Food Safety Authority (mean ± SD).

Micronutrient	All Subjects (n=118)	FB (n=24)	CD (n=23)	CM (n=24)	WM (n=24)	A (n=23)
Vitamin A (µg)	982 ± 995 (131%)	942 ± 1214 (126%)	831 ± 687 (111%)	932 ± 503 (125%)	1315 ± 1553 (175%)	892 ± 521 (119%)
Thiamin (mg/MJ)	0.24 ± 0.08 (240%)	0.24 ± 0.06 (240)	0.24 ± 0.08 (240%)	0.23 ± 0.09 (230%)	0.23 ± 0.08 (230%)	0.25 ± 0.07 (250%)
Riboflavin (mg)	2.5 ± 0.8 (156%)	2.4 ± 0.8 (150%)	2.4 ± 0.7 (150%)	2.4 ± 0.8 (150%)	2.5 ± 0.7 (156%)	2.7 ± 0.9 (169%)
Niacin (mg/MJ)	3.4 ± 0.9 (213%)	3.2 ± 1.0 (200%)	3.7 ± 1.0 (231%)	3.2 ± 0.8 (200%)	3.2 ± 0.9 (200%)	3.7 ± 0.9 (231%)
Pantothenic Acid (µg)	8.2 ± 2.5 (164%)	7.4 ± 3.3 (148%)	8.0 ± 2.1 (160%)	8.5 ± 2.8 (170%)	8.3 ± 1.7 (166%)	8.6 ± 2.5 (172%)
Vitamin B6 (mg)	3.7 ± 1.2 (218%)	3.4 ± 1.1 (243%)	3.6 ± 1.1 (212%)	3.5 ± 1.4 (206%)	3.7 ± 1.0 (218%)	4.1 ± 1.4 (241%)
Biotin (µg)	44 ± 17 (110%)	39 ± 15 (98%)	43 ± 21 (108%)	44 ± 18 (110%)	45 ± 12 (113%)	46 ± 20 (115%)
Folate (µg)	466 ± 177 (141%)	486 ± 216 (147%)	430 ± 136 (130%)	443 ± 194 (134%)	475 ± 140 (144%)	497 ± 190 (151%)
Cobalamin (µg)	7.7 ± 3.1 (193%)	7.1 ± 3.0 (178%)	7.3 ± 2.7 (183%)	7.1 ± 2.1 (178%)	9.0 ± 4.2 (225%)	7.8 ± 2.5 (195%)
Vitamin C (mg)	160 ± 111 (145%)	163 ± 107 (148%)	131 ± 78 (119%)	199 ± 175 (173%)	153 ± 89 (139%)	154 ± 70 (140%)
Vitamin D (µg)	2.5 ± 1.4 (17%)	2.0 ± 0.9 (13%)	2.3 ± 1.2 (15%)	2.5 ± 1.2 (17%)	3.4 ± 2.0[1] (23%)	2.4 ± 1.2 (16%)
Vitamin E (mg)	10.5 ± 4.5 (81%)	9.6 ± 4.9 (74%)	10.2 ± 5.1 (79%)	11.0 ± 4.4 (85%)	11.9 ± 4.1 (92%)	9.4 ± 4.0 (72%)
Calcium (mg)	1289 ± 353 (136%)	1134 ± 276 (119%)	1254 ± 387 (132%)	1363 ± 359 (144%)	1393 ± 338 (147%)	1300 ± 366 (137%)
Iron (mg)	17 ± 5 (155%)	17 ± 5 (155%)	16 ± 4 (145%)	17 ± 6 (155%)	18 ± 5 (164%)	18 ± 6 (164%)
Sodium (mg)	4179 ± 1617 (209%)	3532 ± 1646 (177%)	3822 ± 1325 (191%)	4269 ± 1678 (213%)	4697 ± 1627 (235%)	4579 ± 1596 (229%)
Potassium (mg)	4358 ± 1266 (125%)	4079 ± 1190 (117%)	4123 ± 1205 (118%)	4642 ± 1613 (133%)	4467 ± 1030 (128%)	4474 ± 1222 (128%)
Magnesium (mg)	409 ± 127 (117%)	383 ± 109 (109%)	400 ± 124 (114%)	427 ± 159 (122%)	423 ± 121 (121%)	414 ± 124 (118%)
Phosphorus (mg)	2096 ± 577 (381%)	1859 ± 532 (338%)	2071 ± 534 (377%)	2179 ± 676 (396%)	2217 ± 501 (403%)	2156 ± 602 (392%)
Copper (mg)	1.9 ± 1.0 (119%)	1.9 ± 1.2 (119%)	1.6 ± 0.6 (100%)	2.0 ± 1.1 (125%)	2.1 ± 1.5 (131%)	1.9 ± 0.5 (119%)
Zinc (mg)	16 ± 5 (114%)	16 ± 5 (114%)	16 ± 4 (114%)	16 ± 5 (114%)	18 ± 4 (129%)	17 ± 5 (121%)
Chloride (mg)	6872 ± 2649 (222%)	5857 ± 2695 (189%)	6230 ± 2201 (201%)	6996 ± 2809 (226%)	7780 ± 2627 (251%)	7494 ± 2558 (242%)
Manganese (mg)	4.6 ± 2.1 (153%)	4.4 ± 2.0 (147%)	4.2 ± 1.6 (140%)	4.6 ± 2.7 (153%)	5.1 ± 2.1 (170%)	4.7 ± 2.0 (157%)
Iodine (µg)	214 ± 94 (143%)	197 ± 75 (131%)	229 ± 113 (153%)	204 ± 97 (136%)	226 ± 94 (151%)	213 ± 100 (142%)
Selenium (µg)	79 ± 25 (113%)	70 ± 21 (100%)	81 ± 25 (116%)	78 ± 23 (111%)	84 ± 29 (120%)	84 ± 28 (120%)

A= Attackers/Strikers; CD= Central Defenders; CM= Central Midfielders; FB= Full Bucks; WM= Wide Midfielders

[1]Different from FB ($p < 0.05$)

Table S5: **Basic** characteristics of the five goalkeepers (mean ± SD).

Basic Characteristics	
Age (yrs)	27.0 ± 4.1
Weight (kg)	85.1 ± 5.3
Height (cm)	187.0 ± 4.5
BMI (kg/m^2)	24.4 ± 1.7
Years of Playing Soccer	9.6 ± 2.7
Official Games Played	121 ± 82

Table S6: Macronutrient intake during the 3-day recording period for the five goalkeepers (mean ± SD).

Macronutrient	Average of 3 Days	Day 1	Day 2 (Match Day)	Day 3
Energy (MJ)	11.8 ± 3.2	15.0 ± 6.4	10.3 ± 1.9	10.0 ± 2.5
Energy (kcal)	2820 ± 765	3585 ± 1530	2462 ± 454	2390 ± 598
Energy (Kj/Kg BM)	137 ± 28	174 ± 64	121 ± 21	116 ± 23
CHO (g/Kg BM)	3.7 ± 0.6	5.1 ± 1.6	2.9 ± 1.2	3.0 ± 0.8
Protein (g/Kg BM)	1.7 ± 0.6	1.9 ± 0.7	1.8 ± 0.5	1.4 ± 0.8
Fat (g/Kg BM)	1.2 ± 0.6	1.5 ± 0.9	1.1 ± 0.5	1.1 ± 0.7
% CHO	46 ± 8	50 ± 6	40 ± 13	45 ± 12
% Protein	21 ± 9	20 ± 10	25 ± 7	20 ± 13
% Fat	33 ± 10	30 ± 7	35 ± 14	35 ± 17

Table S7: Average of the 3-day micronutrient intake in the five goalkeepers. The numbers in parentheses indicate the percentage of the Dietary Reference Values by the European Food Safety Authority (mean ± SD).

Micronutrient		Micronutrient	
Vitamin A (µg)	1497 ± 1324 (200%)	Calcium (mg)	1394 ± 353 (147%)
Thiamin (mg/MJ)	0.24 ± 0.16 (240%)	Iron (mg)	15 ± 4 (136%)
Riboflavin (mg)	2.0 ± 0.9 (125%)	Sodium (mg)	3265 ± 571 (163%)
Niacin (mg/MJ)	3.7 ± 2.1 (230%)	Potassium (mg)	3465 ± 1244 (99%)
Pantothenic Acid (µg)	7.3 ± 3.2 (146%)	Magnesium (mg)	333 ± 72 (95%)
Biotin (µg)	36 ± 12 (90%)	Copper (mg)	1.6 ± 0.7 (100%)
Folate (µg)	326 ± 129 (99%)	Zinc (mg)	14 ± 4 (100%)
Cobalamin (µg)	8.2 ± 4.3 (205%)	Chloride (mg)	5224 ± 795 (169%)
Vitamin B6 (mg)	2.8 ± 1.1 (165%)	Phosphorus (mg)	1981 ± 485 (360%)
Vitamin C (mg)	125 ± 145 (114%)	Manganese (mg)	3.3 ± 0.9 (110%)
Vitamin D (µg)	3.5 ± 1.0 (23%)	Iodine (µg)	143 ± 95 (95%)
Vitamin E (mg)	10.4 ± 4.1 (125%)	Selenium (µg)	77 ± 24 (110%)
